# Prepontine cisternal routine for intrathecal targeted drug delivery in craniofacial cancer pain treatment: technical note

**DOI:** 10.1080/10717544.2022.2134507

**Published:** 2022-10-19

**Authors:** Haocheng Zhou, Dong Huang, Dingquan Zou, Junjiao Hu, Xinning Li, Yaping Wang

**Affiliations:** a Department of Pain Management and Anesthesiology, The Second Xiangya Hospital, Central South University, Changsha, Hunan, China; b Department of Pain, The Third Xiangya Hospital and Institute of Pain Medicine, Central South University, Changsha, Hunan, China; c Hunan Key Laboratory of Brain Homeostasis, Central South University, Changsha, Hunan, China; d Department of Radiology, The Second Xiangya Hospital, Central South University, Changsha, Hunan, China

**Keywords:** Prepontine cisternal, intrathecal, craniofacial, cancer pain, technical note

## Abstract

Intrathecal targeted drug delivery provides effective relief for cancer-related pain. However, its validation in management of craniofacial pain remains much less widely practiced, mainly due to the localized diffusion of analgesic agent with current approach. Here, we report our experience of prepontine cisternal routine for placement and implantation of intrathecal targeted drug delivery in two cases of cancer-related craniofacial pain. Lumbar cannulation was applied and the intrathecal catheter tip was positioned at the prepontine cistern under fluoroscopic guidance during the surgical implantation. Postoperative imaging confirmed that the catheter tip was successfully placed in the prepontine cisternal space. Satisfactory control of pain was achieved after intrathecal therapy, with significant reduction of background and breakthrough cancer pain. None obvious complications were observed in this study. Thus, our novel intrathecal routine may provide an alternative option for craniofacial pain caused by tumor, who were insufficiently treated by oral analgesic agents.

## Introduction

Intrathecal targeted drug delivery (ITDD) has become one useful tool in management of cancer-related pain. To guarantee therapeutic effect, the target of ITDD is generally chosen to be closed to the painful region of the corresponding spinal cord segments (Schultz et al., 2020). Given the further distance between the spinal catheter and cranial nerves, sparse evidence supports the validation of ITDD in craniofacial pain (Appelgren et al., 1996; Baker et al., 2007; Hayek et al., 2016; Moman et al., 2019). Anatomic association between all three divisions of the trigeminal nerve may provide the analgesic foundation for intrathecal therapy at high cervical site (Taren and Kahn, 1962; Appelgren et al., 1996; Baker et al., 2007). Compared with upper cervical routine, the cisternal intrathecal access remains one promising yet rarely applied technique in orofacial pain treatment (Narváez et al., 2002; Dupoiron, 2020). One significant advantage of cisternal administration is the concentration of analgesic agents near the cranial nerve (i.e. trigeminal nerve). In our center, ITDD therapy is performed to treat cancer-related craniofacial pain, and we applied one novel prepontine cisternal routine via lumbar puncturing technique. Here, we introduce our experience of this procedure in two cases with intractable cancer craniofacial pain.

## Methods

### Patients

ITDD was performed with prepontine cisternal routine in two patients with orofacial cancer-related pain at The Department of Pain Management and Anesthesiology, The Second Xiangya Hospital, Central South University, Changsha, China. One 35-year-old male was diagnosed with tongue cancer and underwent tumor resection in 2016. Unfortunately, he suffered primary nasopharyngeal tumor and orofacial pain since 2019. The second case presented with orbital cancer pain caused by the pulmonary metastasis two years ago. Both cases reported severe pain in the craniofacial region, with maximal self-reported pain scores (8–10 out of 10 visual analogue scale) at resting or breakthrough pain episodes. The procedure was conducted in accordance with the Declaration of Helsinki and approved by the Ethics Committee of The Second Xiangya Hospital, Central South University.

### Relevant anatomic structures in pain processing

The prepontine cistern is one subarachnoid space located dorsally to the clivus and ventrally to the pons. The prepontine cistern contains two cranial nerves, that is the fifth cranial nerve (trigeminal nerve) and the sixth cranial nerve (abducens nerve). The abducens nerve has been considered to transverse the anterior pontine membrane rather than through the prepontine cistern (Matsuno et al., 1988). The trigeminal nerve leaves the mid-pons anteriorly and then courses across the space of prepontine cistern. Subsequently, the fifth cranial nerve courses through the porus trigeminus and enters the Meckel cave, which forms the trigeminal or Gasserian ganglion. The trigeminal ganglion then separates into ophthalmic, maxillary and mandibular branches, which mainly governs the sensory perception in the region of face and head.

### Intrathecal targeted drug delivery apparatus

One polyurethane catheter of 65-centimeter length (ZS2, Linhwa, China) was applied for the intrathecal drug delivery, which was connected to one implantable port (ZS2, Linhwa, China). To adjust the speed of drug diffusion manually, the implantable port was connected to one external electronical pump (ZZB-150, Aipeng, China) through one plastic tubing. The total volume of analgesic agents was 150 ml, with one velocity of 0.1 ml per hour at initial administration. Interval period of bolus was set 30 minutes with 0.1 ml volume for the rescue of breakthrough pain.

### Surgical technique

**Figure 1.**
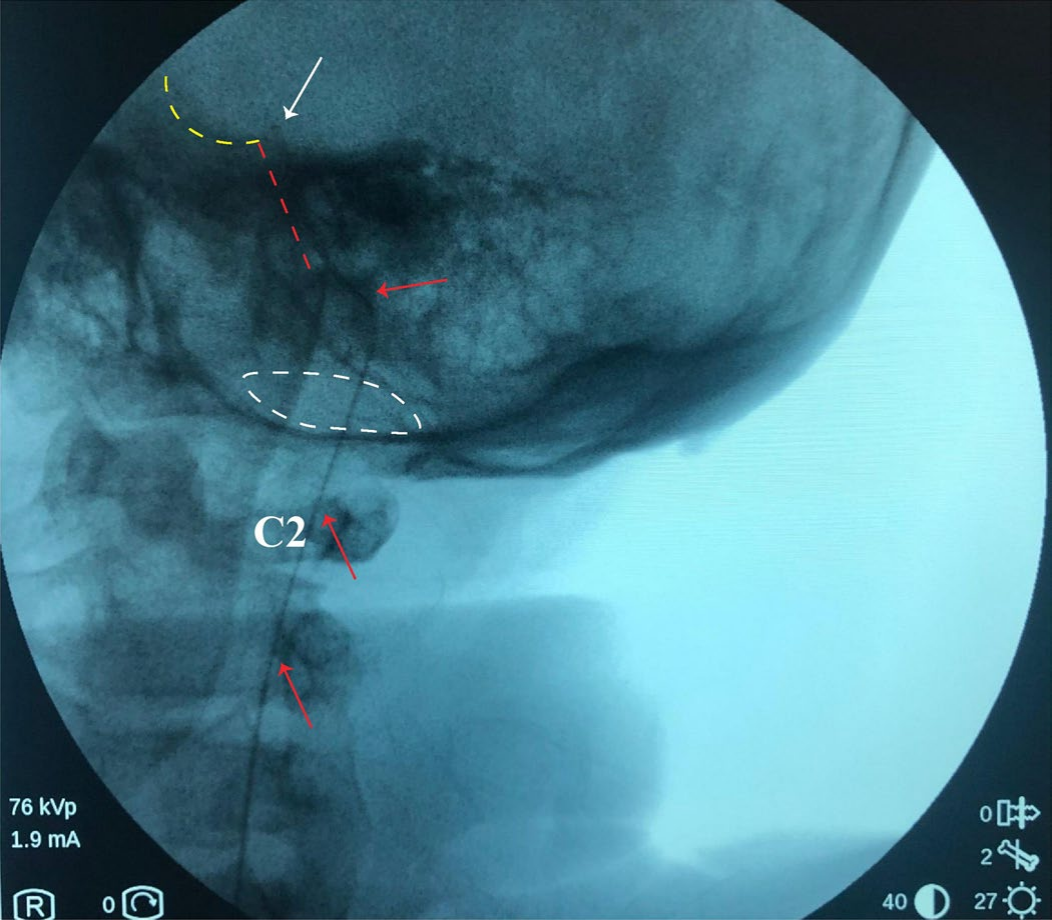
Location of intrathecal catheter tip confirmed by the fluoroscopy during surgery. The red arrow indicates the intrathecal catheter; the white arrow indicates the tip of catheter; white dash cycle indicates the foramen magnum; red dash cycle indicates the clivus; the yellow dash cycle indicates the pituitary fossa.

To avoid discomfort and anxiety during the procedure, general anesthesia was applied and the patient was placed in a left lateral decubitus position. We performed one lumbar puncturing access at the L2/3 intervertebral space with one 14-G Tuohy needle. The cannulation of subarachnoid space was then confirmed by a clear cerebrospinal fluid (CSF) tap. Intrathecal catheter was inserted through the Tuohy cannula and advanced under the guidance of fluoroscopic imaging. The placement of intrathecal catheter into the high cervical space can be confirmed by the lateral view of fluoroscopy, after that we gradually inserted the catheter through the foramen magnum. The tip of catheter is designed to be positioned at the clivus, as shown in the Figure 1. Patient was required to stay in bed for at least two days after procedure, to avoid hypotensive cranial pressure headache caused by CSF leaking.

### Postoperative three-dimensional reconstruction

To confirm the location of catheter tip, three-dimensional reconstruction of computed tomography was performed within one week after surgery. The parameter of digital imaging scanning and imaging processing was similar with our previous study (Wang et al., 2021), helical images were acquired between the lower lumbar segments (L3–L5 level) and the calva line with detector width of 0.625 mm, 120 kVp, and 200 mAs. The raw data were then transferred one imaging workstation (Philips) for the reconstruction for catheter placement.

## Results

### Catheter placement

**Figure 2.**
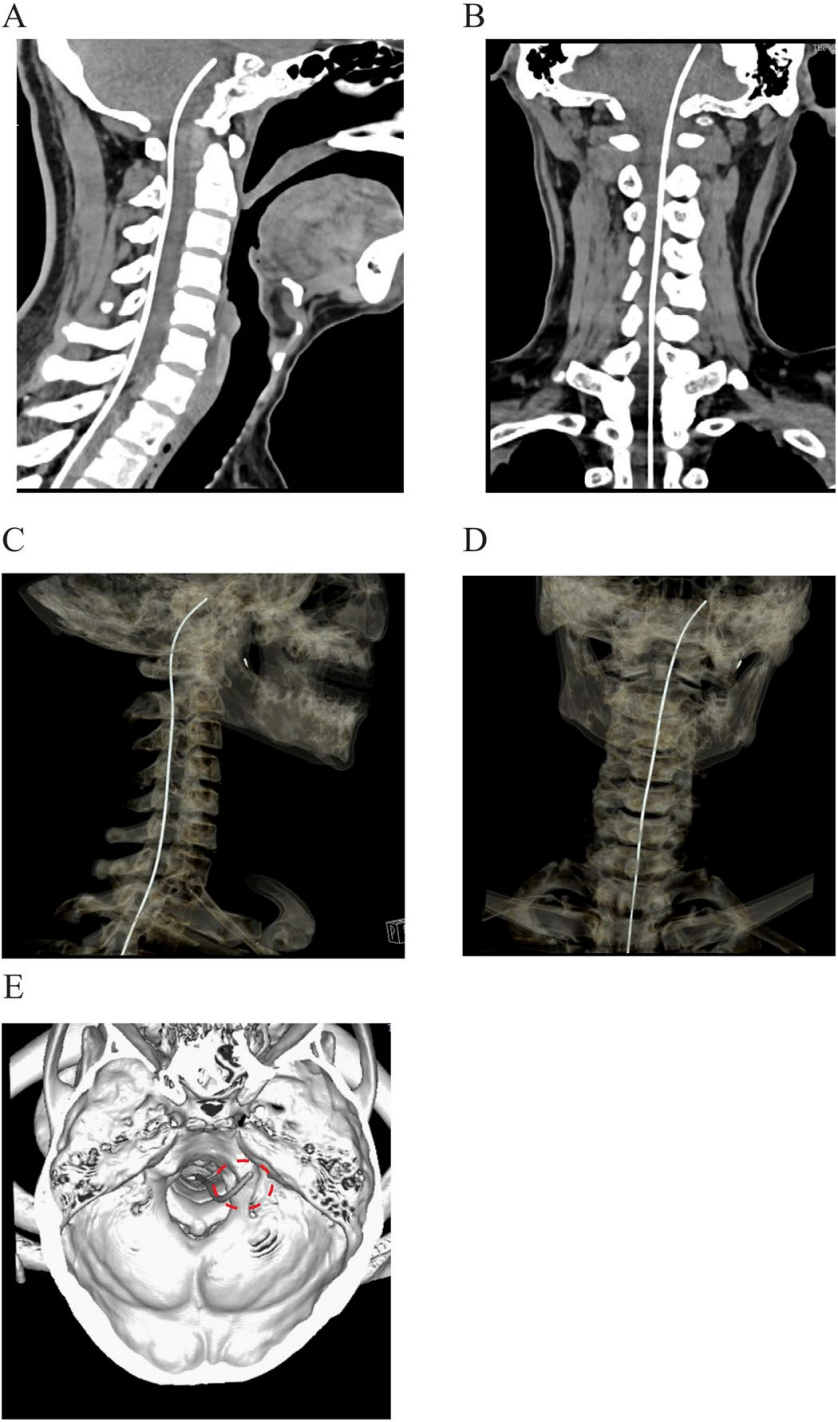
Postoperative imaging of catheter placement with computed tomography. a, b: Sagittal and coronal plane of scanning demonstrated that the tip of intrathecal catheter was located at the level of posterior clinoid process. c, d: Three-dimensional reconstruction of catheter placement by computed tomography. e: Reconstruction of the skull base to reveal the location of the catheter (circled by the red dash line).

Postoperative imaging examination was scheduled before discharge. Two patients did not present any abnormal manifestations during the imaging scanning. The tip of intrathecal catheter was positioned at the level of posterior clinoid process, as shown in the sagittal and coronal plane (Figure 2a,b). In the three-dimensional reconstruction, we can find that the tip of catheter was located unilaterally in the prepontine cisternal space (Figure 2b–d). The implantation sites of catheter tip were consistent with the painful region, approaching to the left hemisphere for the first patient, and right side for the second case respectively.

### Pain severity

Before intrathecal therapy, both patients scored their pain as 8 of 10 VAS at resting stage. Only mild pain was reported in both cases after ITDD treatment, with general pain scores decreasing to 1–3 VAS. Both cases described their breakthrough cancer pain as ‘worst pain’ before surgery, ranking the pain scores as 10 of 10 VAS with more than 20 episodes every day. After surgery, none significant breakthrough pain was reported in the first case, with maximal pain scores below 4/10 VAS. The other case reported 5 to 6 out of 10 VAS breakthrough pain after ITDD implantation, which can be efficiently attenuated by patient-controlled booster.

### Analgesic medication usage

Daily morphine equivalent dose before implantation procedure was 380 and 790 mg respectively. The initial intrathecal morphine dosage of 24-hour was set 0.03 mg according to our previous protocol (Zou et al., 2021). Before the morphine titration was accomplished, oral opioids were administrated according to the preoperative dosage and pain severity. The first patient did not take any oral opioids medication one week after surgery, and the intrathecal dosage of morphine was kept at 1.0–2.4 mg per 24 hours with a 160–380 conversion ratio. The second patient still took oral opioids with initial morphine dosage of 0.24 mg per 24 hours before discharge. The transition of oral to total intrathecal administration was accomplished in one month after procedure. Intrathecal morphine usage was 1.2–3.0 mg (conversion ratio: 260–660) in every 24 hours at one-month follow-up.

### Complications

We did not observe any obvious complications related to the surgical operation in either case (e.g. hemorrhage, paresthesia, respiratory depression, or low-pressure headaches). The first case had fever in the first 24 hours and recovered with physical cooling.

## Discussion

ITDD is one useful tool for management of cancer-related pain. However, its validation in the orofacial region is rarely reported in previous study, despite the urgent need of pain relief in patients with craniofacial neoplasm (Dupoiron, 2020). Recently, we have reported one successful case of orofacial cancer-related pain, who was treated by the ITDD with prepontine cisternal routine (Zou et al., 2021). Given the adjacent position to the cranial nerves, prepontine cisternal routine may provide one promising therapeutic target for ITDD in craniofacial pain caused by tumor. Here in this technical note, we report our experience of prepontine cisternal access for ITDD in two cases with primary or metastatic craniofacial tumor.

The principle of ITDD catheter placement is determined by the most painful location reported by the patient. For example, we routinely perform thoracic ITDD (T5–T8) to treat visceral pain caused by gastrointestinal tumor, which is consistent with previous literature (Pak & Hung, 2019). However, the classical strategy of intrathecal routine is rarely used in treatment of craniofacial pain. Alternatively, high cervical intrathecal or cisternal route has been applied to control facial pain (Dupoiron, 2020). Intrathecal delivery of morphine may target the trigeminal nerve root, lower brain stem and the midbrain, which mainly govern the processing of craniofacial pain signal.

Consistent with spinal cord implantation, one key of cisternal routine is to confirm the location of the catheter tip during the surgery. To guarantee the therapeutic effect, the catheter should be placed into the cisterna magna (Appelgren et al., 1996; Narváez et al., 2002; Lundborg et al., 2009). To our knowledge, it is the first time that we inserted the tip of catheter in to the prepontine cisternal space, which located cephalad to the cisterna magna and ventrally to the pons. Given the flow of CSF, the superiority of this approach over traditional cisternal routine remains uncertain. However, the shorter distance between the catheter and the trigeminal nerve root may improve the infusion of analgesic medication.

In addition to intracisternal depth, we found that the tip of intrathecal catheter was placed toward the painful side in the prepontine cisternal space, as confirmed by the CT scanning (Figure 2). Unlike spinal cord stimulation, the stimulation electrode should be unilaterally placed to corresponding side of pain (Dong et al., 2017; Zhou et al., 2022). In intrathecal drug administration, the analgesic agents can diffuse to the opposite side through the CSF. Thus, we think it is not necessary to insert the catheter tip into the painful side during the surgery. The comparison of analgesic effect between ipsilateral and contralateral position needs to be further investigated in the future study.

In the end stage of tumor, one hallmark feature of cancer-related pain is the excruciating pain, that is insufficiently treated by oral analgesic medications. Likely, both cases presented with severe pain (8/10 VAS) at resting state and the worst suffering during breakthrough pain episodes, with considerable amounts of opioids consuming up to 380 and 790 mg equivalent morphine in 24 hours. Despite unsatisfactory control of pain, multiple side effects of analgesic effect are frequently reported, including dizziness, nausea, vomiting, constipation and physical dependence (Benyamin et al., 2008). One advantage of intrathecal therapy is the significant reduction of opioids intake, which may attenuate the side reaction. Oral opioids were totally replaced one week after implantation procedure in the first patient, and the intrathecal titration was completed one month after discharge for the other case. The daily cisternal amount of morphine ranged between 1.9 and 3.0 mg, accounting for about 0.05% of oral dosage. This data is consistent with the conventional 300:1 ratio (Sylvester et al., 2004). In addition to conversion ratio, one key parameter of intrathecal therapy is the pharmacratic formation. Combination of local anesthetics (bupivacaine) with morphine may contribute to provide supplementary pain control for the intractable cases (van Dongen et al., 1999). In this study, both cases achieved sufficient relief with prepontine cisternal morphine delivery.

One common and life-threatening complication of cisternal administration is the infectious meningitis, especially for those with and tumor (Dupoiron, 2020). We observed that one case had fever within the first 24-hour post-surgery. Given the undernourished and immunosuppressive status in patient with orofacial tumor, we think it necessary to apply the prophylactic antibiotics prior to and after implantation procedure.

## Conclusions

The prepontine cisternal routine of intrathecal drug administration provides a feasible and effective drug diffusion to the trigeminal nerve system, which may be an alternative option for the management of cancer-related orofacial pain. However, it is necessary to conduct prospective study with larger number of enrollments in the future study, to further confirm the safety and clinical efficiency.
